# Non-replicative Integral Membrane Proteins Encoded by Plant Alpha-Like Viruses: Emergence of Diverse Orphan ORFs and Movement Protein Genes

**DOI:** 10.3389/fpls.2017.01820

**Published:** 2017-10-27

**Authors:** Andrey G. Solovyev, Sergey Y. Morozov

**Affiliations:** ^1^A. N. Belozersky Institute of Physico-Chemical Biology, Moscow State University, Moscow, Russia; ^2^Institute of Molecular Medicine, Sechenov First Moscow State Medical University, Moscow, Russia; ^3^Department of Virology, Biological Faculty, Moscow State University, Moscow, Russia

**Keywords:** plant virus, insect virus, evolution, movement protein, membrane protein

## Abstract

Fast accumulation of sequencing data on plant virus genomes and plant transcriptomes demands periodic re-evaluation of current views on the genome evolution of viruses. Here, we substantiate and further detail our previously mostly speculative model on the origin and evolution of triple gene block (TGB) encoding plant virus movement proteins TGB1, TGB2, and TGB3. Recent experimental data on functional competence of transport gene modules consisting of two proteins related to TGB1 and TGB2, as well as sequence analysis data on similarity of TGB2 and TGB3 encoded by a viral genome and virus-like RNAs identified in a plant transcriptomes, suggest that TGB evolution involved events of gene duplication and gene transfer between viruses. In addition, our analysis identified that plant RNA-seq data assembled into RNA virus-like contigs encode a significant variety of hydrophobic proteins. Functions of these orphan proteins are still obscure; however, some of them are obviously related to hydrophobic virion proteins of recently sequenced invertebrate (mostly insect) viruses, therefore supporting the current view on a common origin for many groups of plant and insect RNA-containing viruses. Moreover, these findings may suggest that the function of at least some orphan hydrophobic proteins is to provide plant viruses with the ability to infect insect hosts. In general, our observations emphasize that comparison of RNA virus sequences in a large variety of land plants and algae isolated geographically and ecologically may lead to experimental confirmation of previously purely speculative schemes of evolution of single genes, gene modules, and whole genomes.

## Introduction

Plant viruses often induce significant rearrangements of the endomembrane system in infected cells to facilitate viral multiplication and cell-to-cell movement (Verchot, 2011; Grangeon et al., 2013; Heinlein, 2015). The plant-specific membrane compartment involved in viral intercellular movement is represented by plasmodesmata composed of both endoplasmic reticulum (ER) and plasma membrane domains (Maule et al., 2011; Kragler, 2013; Bayer et al., 2014; Brunkard and Zambryski, 2017). Plant viruses encode movement proteins (MPs) targeting plasmodesmata to enable viral spread in infected plants (Schoelz et al., 2011; Heinlein, 2015).

Membrane MPs have been first identified in two viral transport gene modules, triple gene block (TGB) coding for an RNA-binding helicase TGB1 and two small hydrophobic proteins TGB2 and TGB3 (Morozov Yu et al., 1987, 1989; Forster et al., 1988; Morozov and Solovyev, 2003) and double gene block (DGB) encoding two small polypeptides representing an RNA-binding protein and a membrane protein (Hacker et al., 1992; Stuart et al., 2004; Navarro et al., 2006). These findings indicated that movement gene modules composed of two or more cistrons may encode at least one nucleic acid-binding protein and at least one trans-membrane movement protein, which can be rather small. Further studies revealed that, in some cases, multi-component transport modules do not encode dedicated nucleic acid-binding MP, and this function can be performed by viral capsid proteins, as in RNA-containing viruses of the families Closteroviridae and Potyviridae, which employ their flexuous filamentous virions as a transport form of viral genome (Rodríguez-Cerezo et al., 1997; Roberts et al., 1998; Dolja et al., 2006; Solovyev and Makarov, 2016) and DNA-containing viruses of genus Mastrevirus (family Geminiviridae) and the family Nanoviridae (Mandal, 2010; Fondong, 2013). On the other hand, membrane proteins are always found among MPs of multicomponent viral transport systems.

The potyvirus-encoded 6K2 protein, not considered as a true MP, is a membrane protein required for the formation of replicative vesicles, which contain viral RNA polymerase and are capable of trafficking toward the plasma membrane, association with plasmodesmata, and even translocation to neighboring cells (Grangeon et al., 2013; Patarroyo et al., 2013; Jiang et al., 2015). In a similar manner, the TGB2 and TGB3 proteins of Potato virus X (genus Potexvirus) are involved in formation of specialized membrane structures, which are located at plasmodesmata orifices and represent viral replication sites (Tilsner et al., 2013). Therefore, the data on potyvirus and potexvirus movement show that membrane proteins encoded by these two genera can be involved in coupling viral replication and cell-to-cell movement. The membrane protein p6 encoded by Beet yellows virus (genus Closterovirus) is localized to the ER and required for viral cell-to-cell movement, however, its exact functions are unknown so far (Alzhanova et al., 2000; Peremyslov et al., 2004). Interestingly, Citrus tristeza virus, another closterovirus, codes for an additional unique membrane-bound MP specifically required for virus transport in certain plant hosts (Bak and Folimonova, 2015). This finding gives an example of membrane MP acquisition, which probably provides selective advantages to a virus.

Since viral multi-gene transport modules typically encode membrane proteins, the evolutionary origin and phylogenetic links of such proteins are of great importance for understanding the genesis of currently existing plant virus transport systems, as well as general mechanisms of new gene acquisition by plant virus genomes.

## Origin and Functional Significance of Small Membrane Proteins Encoded by TGB and BMB

Recently, we have proposed a two-step helicase gene-centered scenario of TGB evolutionary origin. According to this model, a gene of progenitor accessory helicase (future TGB1 protein), which could have functions of silencing suppression and viral cell-to-cell movement, first evolved to acquire an overlapping gene for a membrane protein TGB2 facilitating the TGB1 movement function, and then the TGB3 gene emerged in the genomic block consisting of the TGB1 and TGB2 genes (Morozov and Solovyev, 2015). Our new sequence analyses and experimental data provide an additional support for this hypothesis.

In the genome of *Hibiscus green spot virus* (HGSV, genus *Higrevirus*), we have recently identified a novel transport gene module termed ‘binary movement block’ (BMB) encoding two proteins, a TGB1-like helicase BMB1 and a membrane protein BMB2 exhibiting a distant relation to TGB2 proteins (Morozov and Solovyev, 2012, 2015). BMB-like gene modules consisting of two genes were also identified in plant transcriptome RNA-seq data assembled into RNA virus-like contigs [virus-like RNA assemblies (VLRA)] (Morozov and Solovyev, 2015). HGSV BMB1 and BMB2 were found to be necessary and sufficient to mediate viral cell-to-cell movement (Lazareva et al., 2017b), and further studies revealed similarities in mechanisms of intracellular transport of BMB and TGB proteins (Lazareva et al., 2017a). Taking into account a distant relationship of TGB and BMB proteins, we hypothesize that the BMB transport module could represent an evolutionary snapshot of intermediate stage of TGB evolution corresponding to the TGB1/TGB2 genomic block. Additionally, in the transcriptome of *Andrographis paniculata*, we identified a VLRA (Ap-VLRA) with a gene array coding for polypeptides most closely related to proteins encoded by viruses of the genus *Carlavirus* (family Betaflexiviridae) (NCBI accession numbers GBSY01021950, HG5O3SP01E9V5B). In the Ap-VLRA, a gene block consisting of TGB1 and TGB2 is immediately followed by a downstream gene of viral capsid protein, and no TGB3-like gene (or any other ORF) could be identified downstream of the TGB2 gene (data not shown). Moreover, recent sequence analyses of cassava-infecting viruses of the genus *Potexvirus* (family Alphaflexiviridae) revealed that *Cassava virus X* (CsVX) and *Cassava new alphaflexivirus* (CsNAV), both having a gene arrangement typical for potexviruses, lack the TGB3 gene (Lozano et al., 2017). Interestingly, CsVX is rather inefficiently transmitted to *Nicotiana benthamiana*, whereas CsNAV is unable to infect this plant host altogether (Lozano et al., 2017). These data support our earlier hypothesis that TGB3-related gene could be an accessory, rather than essential, TGB transport system component, which may increase the transport efficiency in certain hosts species or plant tissues (Lezzhov et al., 2015; Morozov and Solovyev, 2015). This hypothesis is in agreement with earlier observations that the cell-to-cell transport of *Potato virus X* (PVX; genus *Potexvirus*) can occur in the absence of TGB3, although the efficiency of transport is greatly decreased (Tamai and Meshi, 2001). Likewise, *Alternanthera mosaic virus* (AltMV) containing a premature stop codon in TGB3 gene exhibits a limited transport between adjacent epidermal cells (Lim et al., 2010). Interestingly, while PVX TGB3 has been shown to co-localize with the viral replication complexes (VRCs) at the ER near PD (Tilsner et al., 2013, see above), AltMV TGB3 localizes to chloroplast membranes, which may be the main site of AltMV replication (Lim et al., 2010; Jang et al., 2013). Considering one of the transport models for potexviruses (Park et al., 2014), where RNP movement complex containing the replicase, TGB1 protein, CP, and genomic RNA is released from virus replication sites by TGB3 protein and targeted to PD by TGB3 in association with TGB2, it can be proposed that the significant sequence variations of TGB3 proteins in potex-like TGBs may relate to the diverse compartmentalization and differences in fine structural organization between VRC of different viruses. In the course of adaptation to various hosts, viruses with potex-like TGB might have acquired host-specific dependence of cell-to-cell movement on the type of their VRC. However, our recent experimental data on HGSV BMB indicate that the distant relatives of TGB1 and TGB2 encoded by HGSV – BMB1 and BMB2 – are, rather efficiently, able to support movement of TGB-deficient PVX, although HGSV BMB1 and BMB2, apparently, do not have obvious adaptation to PVX VRC (Lazareva et al., 2017b). Thus, if the need of a tight connection of VRC to movement is not essential for virus multiplication and spread, some viruses like CsVX and CsNAV can move cell-to-cell in their natural hosts using only the activities of TGB1 and TGB2. These data demonstrate that a genomic module consisting of a helicase and a TGB2-related membrane protein is in principle sufficient for viral cell-to-cell movement, validating the proposed two-step scenario of the TGB evolution.

**FIGURE 1:**
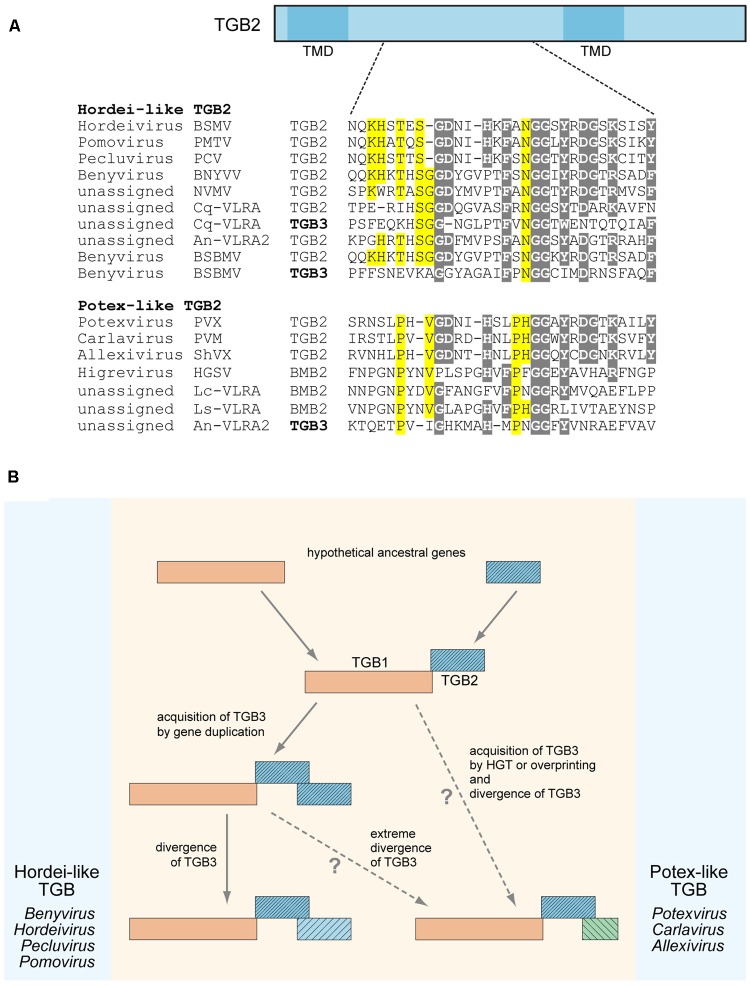
Sequence conservation and possible evolutionary origin of TGB2 and TGB3 proteins. **(A)** Alignment of amino acid sequences located in the central conserved hydrophilic region between two transmembrane domains (TMD) of TGB2 proteins. TGB2 protein is schematically shown above the alignment as a box; the positions of TMDs are indicated; the position of aligned sequence region in TGB2 is shown by dashed lines. TGB3 sequences showing similarity to TGB2 sequences in the aligned region are included into the alignment and shown by bold. Alignments for hordei-like and potex-like TGB2 proteins are presented separately. Gray shading indicates residues conserved in both TGB types, yellow shading shows residues specifically conserved either in potex-like TGB2, or in hordei-like TGB2. BSMV, *Barley stripe mosaic virus*; PSLV, PMTV, *Potato mop-top virus*; BSBV, *Beet soil-borne virus*; PCV, *Peanut clump virus*; BNYVV, *Beet necrotic yellow vein virus*; NVMV, *Nicotiana velutina mosaic virus*; BSBMV, *Beet soil-borne mosaic virus*; PVX, *Potato virus X*; PVM, *Potato virus M*; ShVX, *Shallot virus X*; HGSV, *Hibiscus green spot virus*; CqVLRA, *Colobanthus quitensis* contig 125488 (NCBI accession GCIB01126289); An-VLRA2, *Asplenium nidus* assembly (1KP database accession PSKY-2058768); Lc-VLRA, *Litchi chinensis* assembly (1KP database accession WAXR-2010981); Ls-VLRA, *Lathyrus sativus* (NCBI accession GBSS01016353). **(B)** Proposed general scheme of TGB evolution. Processes specific for potex-like and hordei-like TGBs are shown on the right and left, respectively.

We previously suggested that the TGB3 gene could emerge in the transport module consisting of TGB1 and TGB2 as a result of overprinting, when a coding region became translatable in a different reading frame, horizontal gene transfer (HGT), or duplication of the TGB2 gene and subsequent divergence of the two copies (Morozov and Solovyev, 2015). Our additional sequence analyses shed a new light on these possibilities. In the transcriptome of *Colobanthus quitensis*, we found a VLRA (Cq-VLRA) resembling a plant virus genome fragment encoding TGB proteins related to the respective proteins of the genus *Benyvirus* (**Figure 1A**). *Deschampsia antarctica* (Poaceae) and *Colobanthus quitensis* (Caryophyllaceae) represent the only two vascular plants species in the Antarctica coast flora isolated from the rest of the world for approximately 20 million years (Cantrill and Poole, 2013). Thus it can be proposed that Cq-VLRA encodes an evolutionary early variant of TGB. The central region of Cq-VLRA TGB3 protein located between two transmembrane sequence segments shows sequence similarity to the Cq-VLRA-encoded TGB2 protein, exhibiting conservation of most of the amino acid residues invariant in TGB2 proteins (**Figure 1A**). In a similar manner, the TGB3 protein of *Beet soil-borne mosaic virus* (BSBMV, genus *Benyvirus*) shows a detectable similarity of its central region to TGB2 proteins (**Figure 1A**). These observations indicate that at least in benyviruses the TGB3 gene could arise by a duplication of TGB2 gene.

It should be noted that previously we have distinguished two types of TGB, namely potex-like and hordei-like TGB, which differ in both domain organization of TGB1 and the structure of TGB3 proteins, but have similar TGB2 proteins with two transmembrane domains and a highly conserved central region between them (Morozov and Solovyev, 2003). Among hordei-like TGBs, TGB3 proteins of benyviruses form a distinct group since they are unrelated to other TGB3 proteins (Morozov and Solovyev, 2003; Verchot-Lubicz et al., 2010). In addition, pairwise sequence comparisons of TGB3 central regions revealed that identity between different benyviruses [except the pair *Beet necrotic yellow vein virus* (BSBMV)] is lower that 28%, whereas sequence identity between the genera *Hordeivirus*, *Pomovirus*, and *Pecluvirus* is higher than 33% (data not shown). Thus, benyvirus TGB3 proteins show a high degree of sequence diversity, and some of these proteins have sequence similarity to TGB2 proteins (see above). These observations could indicate that the benyvirus TGB3-encoding genes underwent a considerable divergence after evolutionarily recent duplication of the TGB2 gene.

Surprisingly, the TGB3 protein encoded by an *Asplenium nidus* VLRA exhibits a sequence similarity to TGB2 proteins of potex-like TGB, whereas its TGB2 belongs to the hordei-like type (Morozov and Solovyev, 2015) (**Figure 1A**). This observation might imply that TGB3 gene origin could be a result of HGT from a different virus genome. Thus, both gene duplication and HGT could be considered as possible mechanisms of TGB3 gene origin in the TGB evolution. On the other hand, the absence of any sequence similarity between potex-like and hordei-like TGB3 proteins could indicate that potex-like TGB3 either evolved independently, or emerged as a result of an extreme divergence with hordei-like TGB3 (**Figure 1B**) (Morozov and Solovyev, 2003; Verchot-Lubicz et al., 2010).

## Poorly Characterized Hydrophobic Proteins Encoded by Plant Alpha-Like Viruses

**FIGURE 2:**
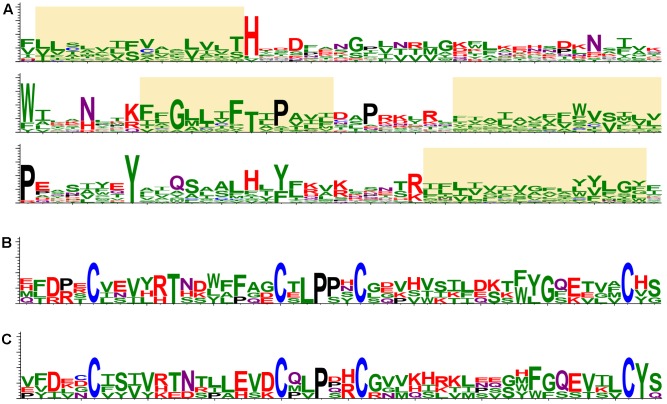
Protein structure conservation depicted as sequence logos based on aligned protein sequences. **(A)** The most conserved region of SP24 proteins in plant and insect viruses. Yellow boxes indicate stretches of hydrophobic amino acid residues. The logo is based on the following sequences: *Loreto virus* (accession number KX518775); *Anopheline-associated C virus* (KF298279); *Blueberry necrotic ring blotch virus* (JN651150); *Chronic bee paralysis virus* (ANG65715); *Hibiscus green spot virus* (HQ852054); *Ambrosia trifida* (GEOH01007094); *Triticum polonicum* (GEDQ01066052); *Camellia sinensis* (GFMV01045386 and GARM01000026); *Paulownia tomentosa* (GEFV01018861); *Panax ginseng* (GDQW01045297); *Elaeocarpus photiniifolius* (FX134396); *Gevuina avellana* (GEAC01035000); *Citrus leprosis virus C* (NC008170); *Humulus lupulus* (GAAW01021049). **(B)** CBPV ORF2-like proteins in insect viruses. The logo is based on the following sequences: *Chronic bee paralysis virus*, *Loreto virus*, *Negev virus*, *Piura virus*, and *Wuhan house centipede virus 1*. **(C)** CBPV ORF2-like proteins in virus-like RNA in plant transcriptomes. The logo is based on the following sequences: *Triticum polonicum* (GEDQ01066052), *Panax ginseng* (GDQW01045299), *Camellia sinensis* (GFMV01053147), and *Talbotia elegans* (SILJ-2021063). The sequence logos visualize the distribution of amino acid residues at each position of conserved motifs. Amino acids are represented in a single-letter code and colored as follows: N and Q, purple; D, E, K, R and H, red; P, black; C, blue; S, T, Y, A, I, L, M, F, V, G and W, green.

Retrospectively, the first “orphan” membrane protein was found in several insect-transmitted plant viruses, namely, cileviruses, higreviruses, blunerviruses (Kuchibhatla et al., 2014). This polypeptide is a member of SP24 protein family (PF16504), which contains a domain corresponding to the central region of the conserved hydrophobic protein of insect chroparaviruses and negeviruses (Shi et al., 2016; Nunes et al., 2017). SP24 family protein is probably one of the major structural components of *Chronic bee paralysis virus* (CBPV) virions (Chevin et al., 2015). We further analyzed potential occurrence of SP24-like sequences in plant viruses using recent transcriptomic databases at NCBI (National Center for Biotechnology Information^1^) and 1KP (The 1KP initiative generated large-scale gene sequencing data for over 1000 species of plants^2^). Interestingly, a dozen of plant virus-like RNA assemblies (VLRAs) encoding SP24-like proteins and having diverse ORF organizations was found. All new plant VLRA-encoded SP24-like proteins are only moderately similar to the previously described plant virus SP24 hydrophobic proteins (**Figure 2A** and Supplementary Figure 1). These data argue that SP24 homolog coding sequence is rather widely distributed among plant viruses. Interestingly, all plant and insect SP24 proteins possess a poorly conserved N-terminal region, which is enriched in positively charged amino acid residues (Kuchibhatla et al., 2014). Since plant cileviruses, higreviruses, blunerviruses, and insect negeviruses encode no typical nucleocapsid proteins (Nunes et al., 2017), we propose that SP24 could be a virion shell component capable of direct interaction with encapsidated viral RNA due to its positively charged N-terminal region. Such a role of this SP24 region could be reminiscent of the function of the positively charged extreme N-terminus of capsid protein in many small icosahedral viruses known to interact with viral RNA to mediate virion assembly and stabilize the resulting structure (Ford et al., 2013; Garmann et al., 2014).

Evidently, surface proteins of insect viruses, similarly to mammalian viruses, are required for movement (entry into and exit from cells) in animal hosts (Zhong et al., 2013). We propose that some plant viruses may have two sets of movement genes required for spread in plants and insect vectors. This can be illustrated by cileviruses and higreviruses. Citrus leprosis virus C RNA2 encodes an MP similar to the 3a MP found in bromoviruses and cucumoviruses (PF00803), as well as two “orphan” hydrophobic polypeptides, namely SP24 and p61, which were proposed to participate in virus spread over insect organism (Kuchibhatla et al., 2014). Blueberry necrotic ring blotch virus (BNRBV) and Hibiscus green spot higrevirus (HGSV) potentially also have two movement systems with different specificities encoded in separate genome segments. BNRBV RNA4 contains a single ORF coding for 3a-like MP, whereas RNA3 codes for two “orphan” hydrophobic polypeptides SP24 and p31 (Quito-Avila et al., 2013). HGSV RNA2 encodes recently experimentally characterized BMB MPs, and RNA3 codes for SP24 and “orphan” hydrophobic proteins p33 and p29 (Melzer et al., 2012; Kuchibhatla et al., 2014; Lazareva et al., 2017b).

Another hydrophobic protein (ORF2 protein) is encoded by CBPV RNA2. This protein showed an obvious similarity to the polypeptides encoded by insect negeviruses; however no similarity to plant virus polypeptides, even those of viruses coding for SP24 homologs, was detected (Kuchibhatla et al., 2014; Nunes et al., 2017). The region with most similarity between the CBPV ORF2 protein and proteins of negeviruses corresponds to 50 amino acid residues in the N-terminal part of ORF2, which contains conserved cysteine residues forming disulfide bridges (**Figure 2B**) and possessing the features typical for a virion glycoprotein (Kuchibhatla et al., 2014). Search of recent transcriptomic data at NCBI and 1KP databases showed that hydrophobic polypeptides with conserved signature of cysteines are also present in plant VLRAs (**Figure 2C**). plants (**Figures 2B,C**). Together with the occurrence of similarly positioned transmembrane segments (data not shown), this suggests a common origin of these proteins in different plants and arthropods.

## Conclusion

Membrane proteins encoded by genes acquired by viral genomes in the course of co-evolution with their hosts can be involved in essential processes such as replication, intercellular movement in plants, and spread in insect vectors. Currently available data suggest that the evolution of such genes could involve events of gene duplication and HGT between genomes of plant viruses, as well as between genomes of plant and insect viruses. These evolutionary events apparently account, together with gene shuffling and divergence, for the current diversity of plant virus genomes. Future studies involving newly sequenced plant virus genomes and plant transcriptomes will undoubtedly unravel a complex picture of a non-collinear evolution of viral genome elements and reveal functions of yet uncharacterized viral genes, including those of integral membrane proteins. Ñonsidering TGB evolution, one suggestion for future experimental directions might be to estimate the impact of VRC compartmentalization and functioning in virus intercellular movement. It is quite important to examine this aspect of TGB-mediated transport in comparative studies of related viruses and even virus isolates diverged because of the geographical separation and host range expansion.

## Author Contributions

AS and SM designed and planned the research, SM performed database searches, AS performed sequence comparisons and prepared figures, AS and SM wrote the manuscript and approved the version to be published.

## Conflict of Interest Statement

The authors declare that the research was conducted in the absence of any commercial or financial relationships that could be construed as a potential conflict of interest.

## Footnotes

1 www.ncbi.nlm.nih.gov

2 www.onekp.com
