# Supplementary material for: Non-replicative Integral Membrane Proteins Encoded by Plant Alpha-Like Viruses: Emergence of Diverse Orphan ORFs and Movement Protein Genes
Source: Front Plant Sci. 2017 Oct 27;8:1820. doi: 10.3389/fpls.2017.01820 (PMC5663686; doi:10.3389/fpls.2017.01820)
Supplement: Supplementary file 1 [file Image_1.PDF]

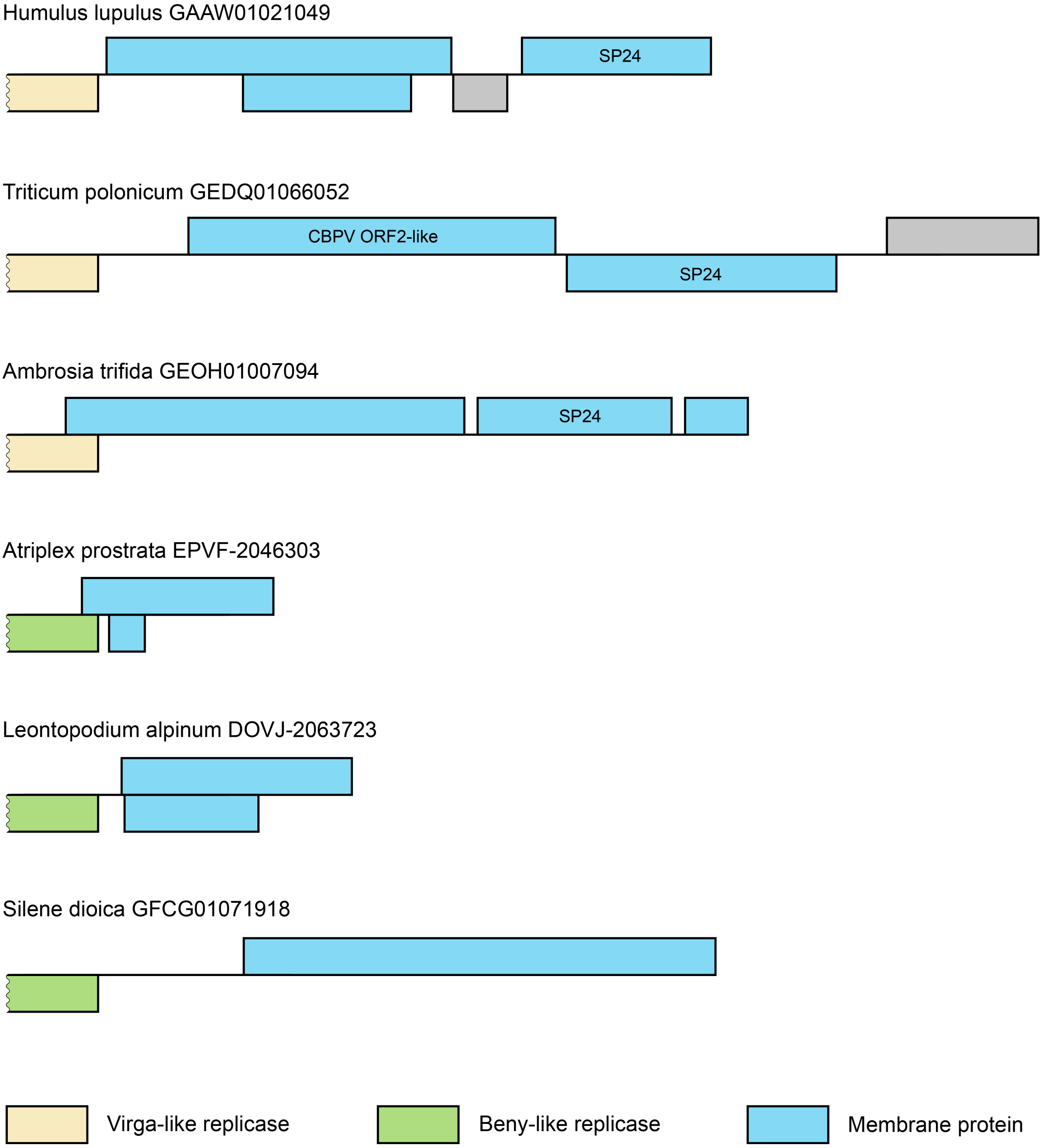

Supplementary Fig. 1. Schematic ORF organization depicting 3'-terminal parts of the putative genomic RNAs found as plant VLRAs encoding virus-like replicases and hydrophobic proteins. Selected plant species and accession numbers are indicated. Replicase proteins and hydrophobic domains are indicated in different colors and abbreviated according to the text.
